# Improvement of Surimi Gel from Frozen-Stored Silver Carp

**DOI:** 10.3390/gels10060374

**Published:** 2024-05-28

**Authors:** Jingyi Yang, Xiliang Yu, Xiuping Dong, Chenxu Yu

**Affiliations:** 1 Department of Agricultural and Biosystems Engineering, Iowa State University, Ames, IA 50011, USA; jingyiy@iastate.edu; 2 Academy of Food Interdisciplinary Science, School of Food Science and Technology, Dalian Polytechnic University, Dalian 116034, China; 3 National Engineering Research Center of Seafood, Collaborative Innovation Center of Seafood Deep Processing, Liaoning Province Collaborative Innovation Center for Marine Food Deep Processing, Dalian 116034, China

**Keywords:** manufactured microfiber, transglutaminase, collagen, response-surface model, heat-induced gel, gel properties

## Abstract

Silver Carp (SC) is an under-utilized, invasive species in North American river systems. In this study, the synergistic effects of manufactured Microfiber (MMF), Transglutaminase (TG), and chicken skin collagen (CLG)) to enhance surimi gel quality from frozen SC were studied. The gel strength, textural properties, rheological properties, water-holding capacity (WHC), water mobility, microstructure, and protein composition of the gel samples were determined to assess the impact of the additives individually and synergistically. The results suggested that TG had the most pronounced effect on the surimi gel properties by promoting protein cross-linking. Synergistic effects between TG, MMF, and CLG can bring effective gel property enhancement larger than the individual effect of each additive alone. With the established response-surface models, the combination of CLG and MMF can be optimized to produce surimi gels with less TG but comparable in properties to that of the optimal result with high TG usage. The findings of this study provided a technical foundation for making high-quality surimi gel products out of frozen-stored SC with synergistic utilization of additives, which could serve as guidelines for the industrial development of new surimi products.

## 1. Introduction

Asian Carps, including Silver Carp (*Hypophthalmichthys molitrix*), Bighead Carp (*Hypophthalmichthys nobilisare*), Black Carp (*Mylopharyngodon piceus*), and Grass Carp (*Ctenopharyngodon Idella*), are invasive species in North America [1]. Referring to their name “Asian,” they are native species in Asian river systems like China and eastern Russia [2]. Silver Carp and Bighead Carp were imported into the U.S. in the 1970s for wastewater treatment [3]. Grass Carp was first introduced into the U.S. as a biological control of aquatic weeds in 1963 [4]. Black Carp was accidentally introduced with Grass Carp in 1973 and then served as biological control of snails [5]. However, these perceived benefits were quickly outweighed by the ecological damages these imported species brought, as the spreading of these fish was poorly managed and the ecological threat of these invasive species was vastly underestimated. Asian Carp are omnivores, feeding easily in river systems with little competition. They grow up and attend larger sizes faster, spread quickly, and are easily out-competing native species [6]. The result is that Asian Carp have grown to a huge population in North American river systems, like the Great Lakes and Mississippi River systems [1,2]. Currently, many institutions and organizations are making an effort to solve this problem. The Asian Carp Regional Coordinating Committee (ACRCC) published the Asian Carp Control Action Plan in 2016 and called for more attention and a focused effort on this problem. Part of the reason why a solution is not easy to come by is that Asian Carp, as it is foreign to the American diet, is under-utilized as a food resource. Hence, their capturing and utilization are not boosted by strong economic incentives. Hence, it is important to find a way to make Asian Carp capturing more robust by generating market-needed products from Asian Carp to create more economic incentives.

Surimi is a gel-like protein-rich food that is very popular in East Asian countries like China, Korea, and Japan. Although surimi is not as popular now in Europe and North America as in Asia, the trend is increasing and it has been on an ascending path for the last decade. Nowadays, it has become much more popular, especially among young, more health/nutrition-aware consumers. Surimi gel is a thermally formed protein gel that is fully cooked, hence microbiological safety is not an issue in surimi products as long as they are produced following HACCP protocols and properly monitored for safety.

Surimi is usually made with deboned marine fish mince. Various surimi products, including fish balls, fish cakes, fish sausage, and imitated crab sticks, are among the most popular surimi products in the market [7]. However, the overfishing of marine fish is decreasing the available resources for surimi production. Currently on the market is imitation crab meat, which is mostly made from fresh-caught marine fish. Finding better ways to make good-quality surimi products from invasive species such as Asian Carp would certainly lower the cost of surimi products (without sacrificing the quality, of course) and would be helpful in making them more attractive to consumers. 

Asian Carp has been widely used to make surimi products in China [7]. However, the surimi products made from Asian Carp are of subpar quality due to limitations posed by Asian Carp themselves. Typically, surimi gel is produced when myofibrillar proteins (the main proteins in fish mince [8]) are irreversibly aggregated and cross-linked through a heat-induced process [9,10]. Asian Carp, such as Silver Carp (SC), are much more easily affected by the “modori” phenomenon (i.e., gel degradation during the heating process), which causes their myofibrillar proteins, especially myosin, to have poorer gelation properties than other fish [11,12]. Moreover, most surimi is made from fresh fish, meaning huge storage costs and processing time limitations. Using frozen storage to extend the processing period will lead to a negative effect on gel formation due to the degradation of myofibrillar proteins [13,14]. Jia’s group also found that the water-holding capacity (WHC) of surimi gel decreased with freezing treatment [15]. Quality improvement methods are needed for high-quality surimi gel production from Frozen Silver Carp (FSC). 

Cryoprotectants are added to surimi made with frozen materials to improve the gel quality [16]. Manufactured Microfiber (MMF), which is cellulosic fiber with a length of a few micron meters and a width of a few hundred nanometers, is an advanced cryoprotective medium [17,18] that can also improve the mechanical properties [19,20] and WHC (i.e., a 72.87% increase at 10 wt% MMF compared to the control in heat-induced chicken salt-soluble meat protein gel) [21] and protect the frozen-stored surimi gel’s structure [22]. MMF also has health benefits since its major content is cellulose, which is widely used as a dietary fiber and benefits the gastrointestinal tract (e.g., a prebiotic effect and bowel movement) [23,24]. Transglutaminase (TG) has been used to improve surimi gel’s textural properties and cross-linking performance in meat products for a long time [25,26] and is widely used in the meat industry as “meat glue”. A collagen extract, a protein-based additive, can enhance the cross-linking process [27] between proteins and has been used for surimi gel improvement [28]. Numerous dietary collagens are by-products of the meat industry (e.g., chicken skin collagen (CLG)) that can lower the cost of the additives used for gel quality enhancement. These additives can be used alone or combined. Our key hypothesis in this study is that the synergistic effects of MMF, TG, and CLG could provide a means to produce surimi from FSC with enhanced properties. Yet, the mechanisms of each of these enhancers on the gel properties need to be understood, and the synergistic effects need to be studied in order to control the processes better so that products with desirable properties can be produced.

In this study, the synergistic effects of MMF, TG, and CLG are evaluated using multiple parameters, including gel strength, texture profile analysis (TPA) properties (e.g., hardness, springiness, and cohesiveness), rheological properties (via the small-amplitude oscillatory shear method (SAOS)), WHC, water distribution, mobility, the myosin cross-linking mechanism (via sodium dodecyl sulfate-polyacrylamide gel electrophoresis (SDS-PAGE)), and microstructures. The Box–Behnken Design (BBD) was used for the experimental design to evaluate the inter-dependences among three additives and produce sufficient data for Response-Surface Modeling (RSM) for process optimization [29,30]. With the application of RSM, the individual effects of each additive and their interdependence on each other at different levels can be analyzed by the estimated quadratic polynomial functions. The purpose of this study was to provide a technical and scientific foundation for using additives in synergistic ways to improve the quality of surimi gel made from frozen SC. It is believed that the methods developed in this study can be easily applied to other Asian Carp species, and ultimately, we aim to provide guidelines for the utilization of Asian Carp in value-added product development for the food industry.

## 2. Results and Discussion

### 2.1. Gel Strength and Textural Properties of the Gel Samples as Affected by the Additives

Gel strength is a useful value to determine the gel quality of surimi gel, which considers the effect of breaking force and deformation distance [31]. The measured textural properties (i.e., TPA parameters) include hardness, cohesiveness, springiness, resilience, and chewiness [32,33]. Hardness shows the required force to break the gel; cohesiveness shows how the surimi withstands the second deformation after the first one; springiness refers to the ability of the gel to spring back after deformation with a waiting period; and resilience denotes the resistance against the gel going back to its original height after the deformation without a waiting period. The multiplication of hardness × cohesiveness × springiness gives chewiness, assessing the easiness of chewing the surimi in the human mouth [34]. Generally, higher values of gel strength and texture properties indicate stronger gel and the changing trends of textural properties are usually consistent with the gel strength results [31,32]. 

For the MMF-only group, there was no significant difference among deformation distances (Table S1), but an increasing trend for breaking force was observed, which led to an increasing trend for the gel strength values as the MMF dosage increased. Specifically, adding 0.5 wt% and 1.0 wt% MMF significantly increased the gel strength by 73.33% and 99.05%, respectively, compared to the control. The 0.5 wt% MMF sample showed the highest hardness (i.e., a 29.76% increase) and chewiness (i.e., a 24.22% increase). These results showed that adding MMF improved the strength of the FSC surimi gel, and this effect was not proportionally dosage-dependent. An optimal dosage exists for the effect of MMF, which appeared to be ~0.5 wt%. 

For the TG-only group, the breaking force significantly increased with the increasing TG dosage. As shown in Table S2, the deformation distances did not change significantly, though. The gel strength had an increasing trend with an increasing TG wt% in a dosage-dependent manner, with the highest value obtained at 1.0 wt% TG (i.e., a 279.21% increase). In comparison, earlier reports on the improvement of gel strength of surimi gel made from frozen-stored longtail southern cod induced by microbial transglutaminase showed a ~200% increase in gel strength at the optimal enzyme dosage (300 U/kg fish), similar to what was observed in this study [31,35].

There were no significant differences in springiness and resilience for the TG-only group. The hardness and chewiness showed an increasing trend, although no significant change existed between 0.1 wt% and 0.5 wt% TG treatments. Adding TG significantly increased the cohesiveness of the gel samples compared to the control, but there were no significant differences among TG treatments with different dosages. The addition of 1.0 wt% TG increased hardness by 206.14% and chewiness by 673.58% compared to the control. These results suggested that the addition of TG led to the formation of surimi gel with much stronger resistance against external forces, which underlined the much higher hardness, cohesiveness, and chewiness. As TG plays its role by catalyzing protein cross-linking formation via covalent bonds, higher TG led to a higher degree of cross-linking formation, which resulted in much stronger network structures in the gel. TG also had a greater impact on the surimi gel than MMF at the same levels. It is reasoned that MMF strengthens the cross-linking protein network via either physical support or hydrogen bonding with proteins, which would not be as effective as the TG. To make strong surimi gel for products such as fish cake, TG would be the most effective additive.

TG is an expensive additive. Hence, it would be more cost-effective if its usage could be partially replaced by other additives. The combined effects of MMF and TG were investigated to see whether the enhancing effects of the TG can be partially substituted by the less expensive MMF. As shown in Table S3, the control group here was 0.5 wt% TG, and the dosage of MMF was increased for each treatment group with a constant 0.5 wt% TG. There was an increasing trend for the breaking force and gel strength value, and the 1.0 wt% MMF and 0.5 wt% TG group had the significantly highest value for breaking force (i.e., a 78.39% increase) and gel strength (i.e., a 96.06% increase) compared to the 0.5 wt% TG control samples. The deformation distance again did not change significantly. Adding MMF to 0.5 wt% TG treatments significantly increased the hardness and chewiness. The hardness of adding 0.5 wt% MMF and 1.0 wt% MMF were significantly larger than adding 0.1 wt% MMF, but there was no significant difference between adding 0.5 wt% MMF and 1.0 wt% MMF. The 0.5 wt% MMF with 0.5 wt% TG group had the highest hardness (i.e., a 120.22% increase) and chewiness (i.e., a 165.39% increase) compared to the 0.5 wt% TG sample. It increased hardness by 258.33% and chewiness by 815.09% compared to the no-additives sample. There were no significant changes in cohesiveness, springiness, and resilience. These results showed that MMF and TG synergistically enhanced the FSC surimi’s gel strength and textural properties of hardness and chewiness. The 0.5 wt% MMF with 0.5 wt% TG sample had higher gel strength, hardness, and chewiness values than those of the 1.0 wt% MMF-only sample and the 1.0 wt% TG-only sample. This result suggested that adding MMF can achieve high-strength surimi gel with lower TG content. Since MMF is more cost-effective than TG and has health benefits [23], the synergistic effect of MMF and TG can be utilized to improve and add value to the FSC surimi products.

Table 1 shows the results of BBD experiments (i.e., MMF, TG, and CLG combinations) for FSC surimi. A third additive, CLG, was used in addition to MMF and TG. CLG can enhance the protein cross-linking process [27] to further improve the gel quality. The results showed that increasing TG wt% improved gel strength, hardness, resilience, and springiness, which is consistent with the results of the TG-only group. There were no significant differences for most samples on cohesiveness. However, treatments no.3 and no.4 (i.e., TG 1% and CLG 3%) had significantly lower cohesiveness, showing that 1 wt% TG with 3 wt% CLG might inhibit FSC surimi to withstand the second deformation. To better understand the synergistic effect of the MMF, TG, and CLG combinations, these data were used to construct response-surface models. As discussed before, higher gel strength and texture properties indicate a stronger gel. A strong gel is generally desirable for its superior processing capability in making products such as fish cakes. However, it is not always the case that the strongest gel would be favored from a consumer perspective. The texture properties affect the sensory attributes of the gel product. If the food is too hard or too chewy, it would require much force to break and becomes uncomfortable and unacceptable for many customers [34]. Hence, it is important to optimize the levels of additives to obtain the appropriate gel strength and textural properties, which are discussed in Section 2.7.

### 2.2. Rheological Properties of the Gel Samples Affected by the Additives

**Figure 1:**
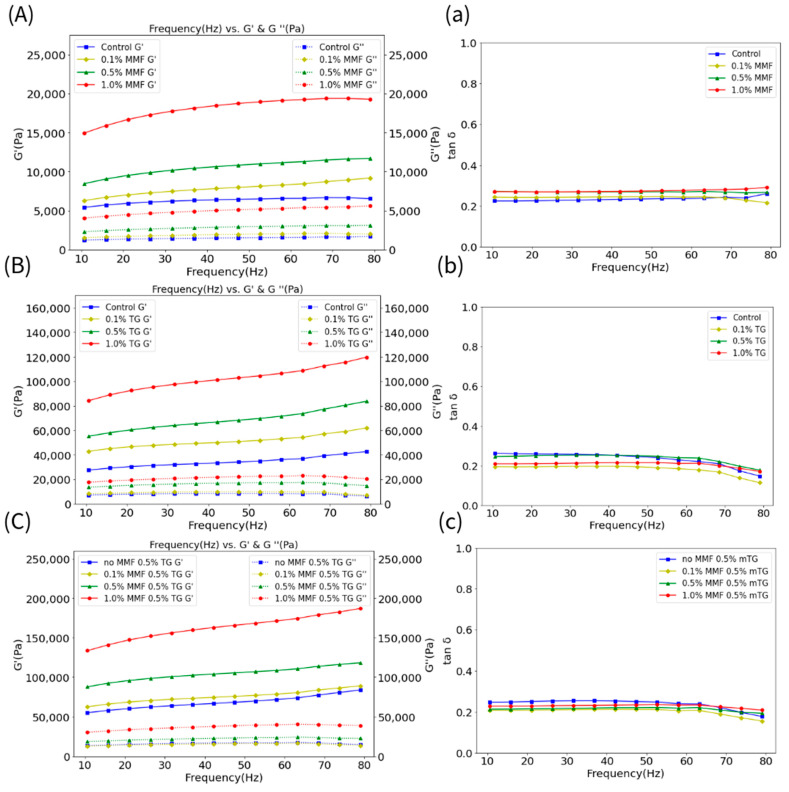
(**A**–**C**) The storage modulus (G′) (solid line) and loss modulus (G″) (dash line) of the MMF−only, TG−only, and MMF–TG combination groups. (**a**–**c**) The tan б of the MMF−only, TG−only, and MMF–TG combination groups.

Figure 1A–C shows the storage modulus (G′) and loss modulus (G″) of the MMF-only, TG-only, and MMF–TG combined groups. Figure 1a–c shows the phase angles (i.e., tan δ = G″/G′). For all groups, G’ was consistently greater than their respective G″, indicating that these samples gelled well and had solid-like behavior. In addition, G′ and G″ values were stable for all samples, showing only slight changes as the frequency increased, indicating a well-formed three-dimensional network structure in the FSC surimi gels. Specifically, the increasing dosage of MMF, TG, and MMF with constant TG led to increasing levels of G′ and G″. The TG-only group had larger G′ and G″ values than that of the MMF-only group, suggesting that TG was more effective in inducing the formation of strong cross-linking networks in the FSC surimi gel than MMF at the same dosage, consistent with the results obtained from gel strength and structural analyses discussed in Section 2.1. The G′ and G″ levels (i.e., 87,000–118,000 Pa and 18,000–24,000 Pa) of the 0.5 wt% MMF with 0.5 wt% TG group were much larger than those of the 1.0 wt% MMF-only sample (i.e., 15,000–19,000 Pa and 4000–6000 Pa) and similar to the 1.0 wt% TG-only sample (i.e., 84,000–120,000 Pa and 18,000–23,000 Pa), showing that there were synergistic effects between MMF and TG to enhance FSC surimi gel, and that the partial substitution of MMF with TG can achieve a comparable rheological effect.

### 2.3. Fourier Transform Infrared Spectroscopy (FT-IR) Results of the Gel Samples

**Figure 2:**
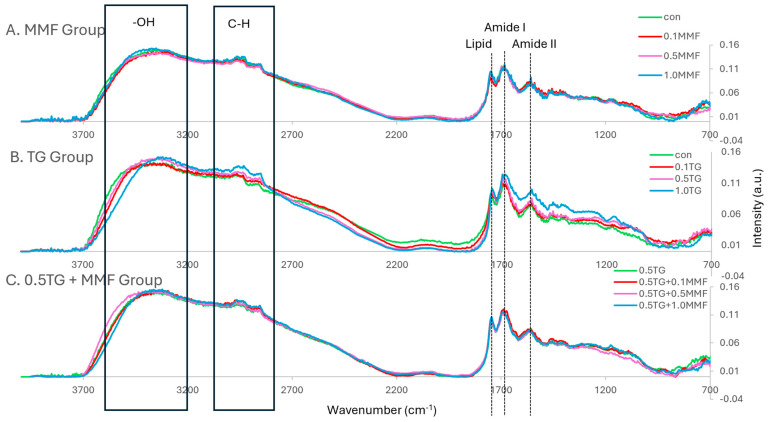
(**A**–**C**) The normalized FT–IR spectra of MMF–only, TG–only, and MMF–TG combination groups.

FT-IR spectroscopy can be used to identify the types of chemical bonds and the interactions of protein molecules in surimi gels [36]. Generally, the characteristic protein bands are amide I (~1655 cm^−1^) and amide II (~1545 cm^−1^) [37]. In surimi gels, the bands for O-H bending (3000–3700 cm^−1^), C-H stretching (2800–3000 cm^−1^), and carbonyl groups of lipids (~1745 cm^−1^) were all strong. As shown in Figure 2A, there was no obvious difference in the spectra with the addition of MMF. In Figure 2B, the addition of TG caused a shift in the O-H bending band to lower wavenumbers and an increase in peak intensities of the C-H stretching band, amide I, and amide II bands. The shift of the O-H bending band suggested that new hydrogen bonds were formed between surimi proteins [36]. The higher intensity of the C-H stretching band, amide I, and amide II peaks suggested more protein content per unit volume of samples [37], indicating a denser protein structure. The samples shown in Figure 2C also showed no obvious differences caused by the addition of the MMF/TG combo. These results revealed that TG had a stronger effect on the interactions of surimi protein molecules than MMF. The effects of MMF and the synergistic effects between MMF and TG might be too weak to be revealed in FT-IR spectra based on the findings in Section 2.1 and Section 2.2.

### 2.4. Water Holding Capacity and Water Mobility of the Gel Samples

**Figure 3:**
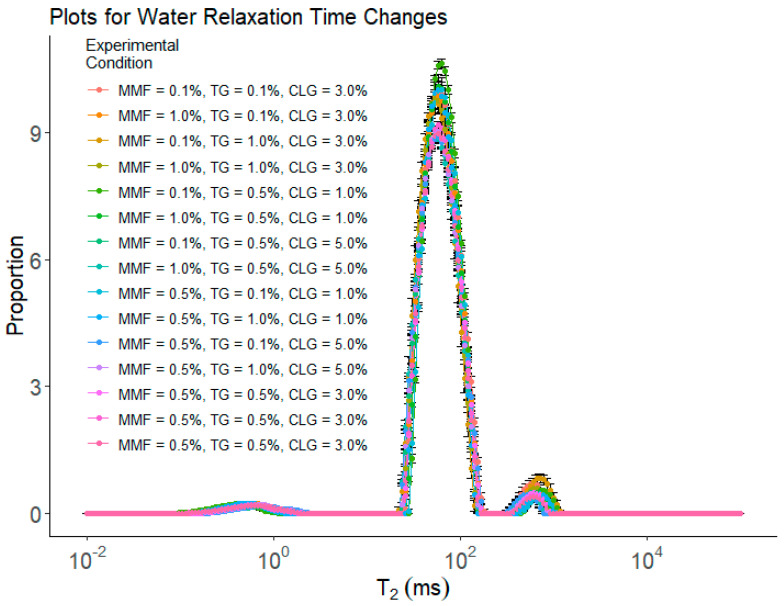
The results of water mobility measured by LF−NMR for BBD treatments. Each treatment had 5 replications (*n* = 5).

Figure 3 and Table 2 show the results of water mobility measured by low-field nuclear magnetic resonance (LF-NMR) for the BBD experiments. All samples had three peaks: 0–1 ms represented the “bound” water (with the lowest mobility) peak (i.e., T_21_); 10–100 ms represented the “immobilized” water (with reduced mobility) peak (i.e., T_22_); and 100–1000 ms represented the “free” water peak (i.e., T_23_). The area proportion for each peak is denoted by P_1_, P_2_, and P_3_, respectively. The X-axis (T_2_) is the log-transformed water relaxation time. The lower the T_2_ value, the stronger the force holding water molecules and limiting their mobility [38]. The Y-axis is the amplitude per unit mass, calculated by Amplitude/Surimi mass. A significant decrease in the ability to hold bound water (i.e., a right shift of the T_21_ peak) in the samples occurred when CLG wt% was increased to 5.0%. The ability to hold immobilized water in the samples grew stronger (i.e., a left shift of the T_22_ peak) when increasing either TG wt% or CLG wt%. The MMP appeared to have a significant effect on the ability of the sample to hold immobilized water when TG was at 0.5 wt%. On the other hand, the ability of the sample to hold free water became weaker (i.e., a right-shift of the T_23_ peak) with increasing TG wt% and became stronger (i.e., a left-shift of the T_23_ peak) with increasing MMF wt% or CLG wt%. As to the amount of water in each category denoted by the values of the Ps, there were no significant changes in bound water among the samples. Treatment no.3 (i.e., MMF 0.1%, TG 1.0%, and CLG 3.0%) had the lowest immobilized water (i.e., P_2_) and the highest free water (i.e., P_3_), while others did not show significant change. It is worth noting that these were samples with the lowest cohesiveness. The underlying correlations between these observations could be associated with the microstructures of the samples, as free water exists mainly in bigger holes/cracks within the gel structure, and these holes/cracks may lead to less resistance of the gel samples against the second deformation applied in cohesiveness tests. The data also showed that, at high TG wt% (1.0%), increasing MMF wt% would result in the transfer of free water to immobilized water.

These results indicated that the addition of additives could alter the way water exists in the gel network. Apparently, the addition of TG and/or CLG both improved the ability of the sample to hold immobilized water, as they either promote protein cross-linking (TG), which may form small 3D traps to hold water inside and lower its mobility (i.e., immobilized water) or are building blocks themselves for the protein cross-linking network (CLG) and enhanced the ability of the sample to trap water molecules and reduce their mobility. MMF seemed only effective in lowering water mobility in the presence of TG. It was reasoned that with stronger protein cross-linking promoted by TG, MMFs were distributed inside the cross-linked network, and their presence further hindered the movement of water molecules in the 3D traps and further reduced their mobility. There was a good example of the synergistic effect: without TG, MMF seemed ineffective in altering water mobility; once TG was added to the system, MMF further enhanced the function of the TG and led to a greater impact on water mobility. 

Furthermore, two separate yet related factors were reported in LF-NMR: The position of the peaks (i.e., the T_2_) and the area of the peaks (the Ps). The T_2_ measures the mobility of water molecules: the lower the T_2_ for each type of water molecule, the lower their mobility [38]. The Ps measure the relative amount of water molecules in each category: the larger the *p* value for a peak, the more water molecules in this category [38]. These two factors are both important in determining how the water exists in a sample. For instance, a left-shifted T_23_ peak with higher P suggests that in this sample, it has a higher portion of free water, which usually is trapped in bigger holes and/or cracks in the sample, but on average, the free water has slightly reduced mobility (which could be due to the spatial hindrance of the cross-linking gel network).

Water mobility could be a factor that plays an important role in changing the water-holding capacity (WHC) of the surimi gel samples. Higher water-holding capacity usually means higher juiciness of the food and lower cooking loss, which are usually more desirable [31]. Lower water mobility (i.e., a greater portion of water exists as immobilized/bound water) means water is more difficult to remove [38], which, in theory, will lead to higher water-holding capacity. However, since water mobility is measured with two factors, as discussed above, their correlations to WHC are usually not straightforward. 

**Figure 4:**
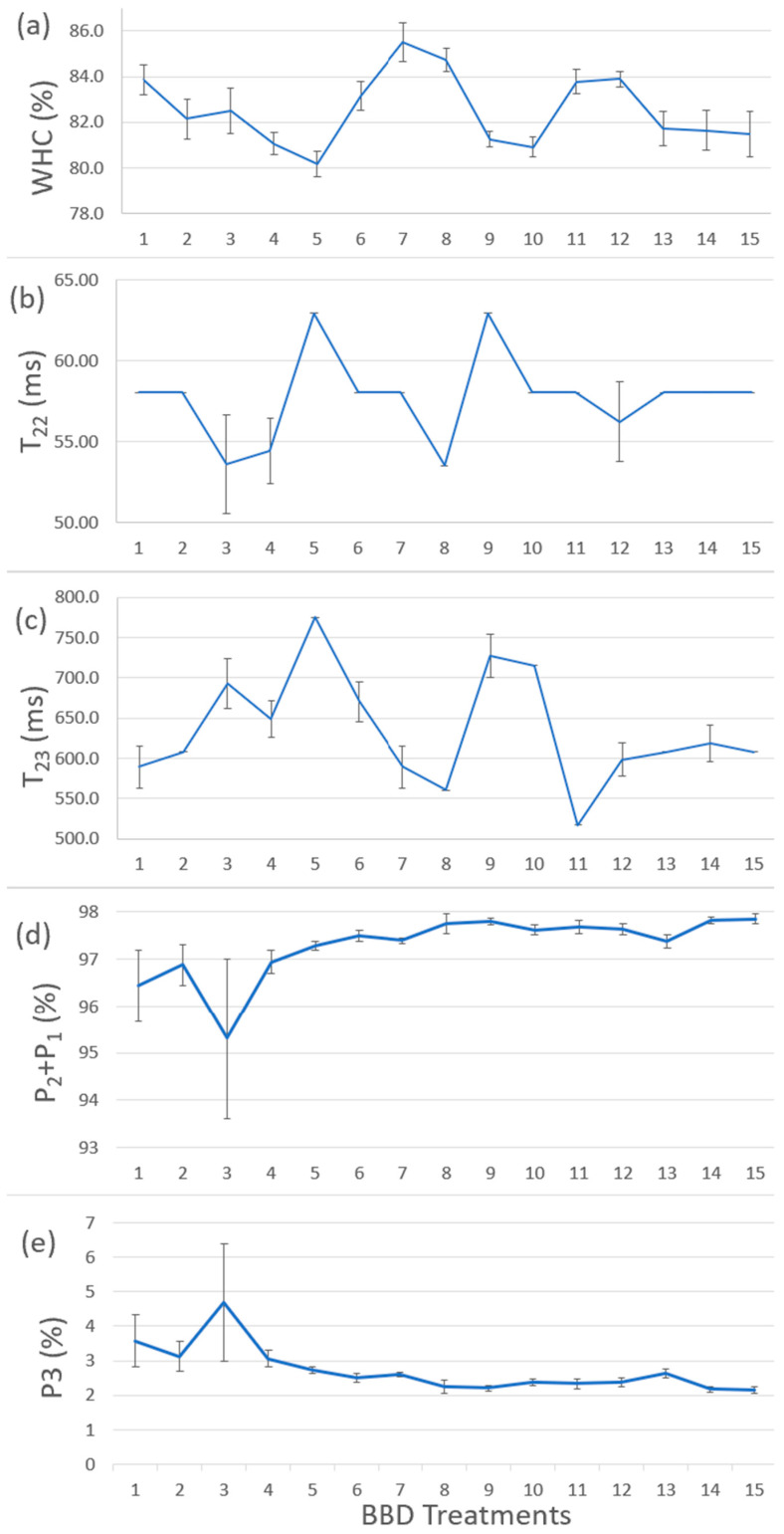
(**a**) The results of WHC for BBD treatments. (**b**) The T22 results from LF-NMR test. (**c**) The T23 results from LF-NMR test. (**d**) The P1 + P2 results from LF-NMR test. (**e**) The P3 results of LF-NMR test. The replication number was 5 (*n* = 5).

Figure 4a shows the results of WHC for BBD treatments. Figure 4b–e shows the T_22_, T_23_, P_1_ + P_2_, and P_3_ values from Table 2. By comparing Figure 4a–e, the samples with larger WHC corresponded to the relatively stronger power to hold immobilized water and free water, indicating that our WHC results were consistent with LF-NMR results. The water content proportion (P_1_ + P_2_ and P_3_) showed slight changes and did not have a comparable trend with WHC results, indicating less or no correlation between WHC and water content proportion in the BBD treatments. Treatments no.7 and no.8 (i.e., TG 0.5% and CLG 5% treatments) had the best WHC value (i.e., 85.50% and 84.74%). Increasing CLG wt% had a stronger effect on enhancing WHC values, while MMF wt% and TG wt% did not have a significant effect. It suggested that CLG had a stronger effect on improving the ability of the protein cross-linking network to trap water molecules and reduce their mobility and, in turn, improve the WHC of the surimi gel.

### 2.5. Microstructural Analysis of the Gel Samples as Affected by the Additives

**Figure 5:**
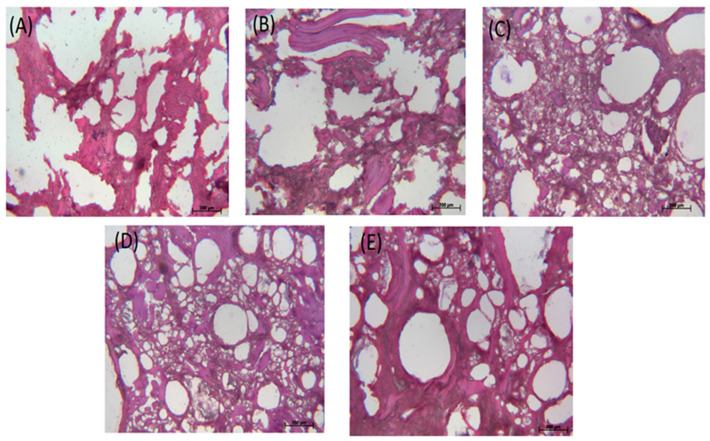
Optical microscopic images of FSC surimi gel. (**A**) Control; (**B**) with 1.0 wt% MMF; (**C**) with 0.5 wt% TG; (**D**) with 0.5 wt% MMF and 0.5 wt% TG; (**E**) with 1.0 wt% MMF and 0.5 wt% TG. The standard scale in the image is 200 µm.

Figure 5A–E shows the optical microscopic images of the control sample, the sample with 1.0 wt% MMF, the sample with 0.5 wt% TG, the sample with 0.5 wt% MMF and 0.5 wt% TG, and the sample with 1.0 wt% MMF and 0.5 wt% TG, respectively. Table 3 shows the pore area analysis and WHC according to the optical microscopic images (Figure S1 shows the binary-processed optical microscopic images for the 0.5 wt% TG sample, for example). It was obvious that MMF and TG improved the network of the FSC surimi microstructure separately and synergistically. There were no large changes in the total pore area in samples with additives, but in all cases, the addition of additives significantly decreased the total pore area compared to the control (i.e., a 22.2–29.3% decrease), indicating that the microstructure changes with additives. The number of pores significantly increased with TG addition (i.e., 360% more than the control), while MMF did not have a significant effect, indicating that TG improved the cross-linking network much better than MMF. With the much-increased pore numbers alongside reduced total pore area, the average pore size in samples treated with TG was significantly reduced, indicating a much more extensive and refined cross-linking network. The samples with TG had relatively larger WHC values than the control and MMF samples, meaning that a TG-enhanced microstructure with smaller yet more numerous pores increased the ability to hold water in these FSC surimi samples. In addition, the synergistic effect of TG and MMF was also demonstrated. The sample with 0.5 wt% MMF + 0.5 wt% TG had a small average pore size, large pore counts, and a better cross-linking structure in the images, revealing that this combination improved the gel quality better than other treatments, which is consistent with the previous findings.

**Figure 6:**
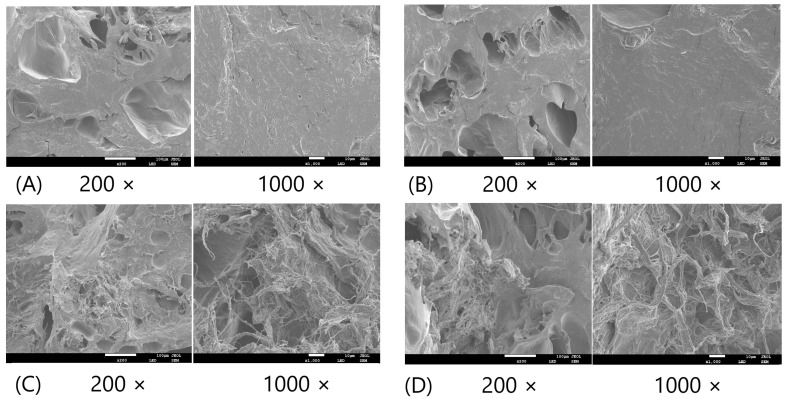
The SEM images of BBD treatments 1–4. (**A**) Treatment 1 (0.1% MMF, 0.1% TG, 3.0% CLG); (**B**) Treatment 2 (1.0% MMF, 0.1% TG, 3.0% CLG); (**C**) Treatment 3 (0.1% MMF, 1.0% TG, 3.0% CLG); (**D**) Treatment 4 (1.0% MMF, 1.0% TG, 3.0% CLG). The standard scale of 200 × image is 100 µm. The standard scale of 1000 × image is 10 µm.

Figure 6 shows the scanning electron microscope (SEM) images of BBD treatments 1–4. Figure S2 provides more images for other BBD treatments. The SEM images confirmed that increasing the TG wt% improved the cross-linking network in the microstructure of FSC surimi gel, consistent with the findings from the optical microscopic images. The increase in MMF and CLG wt% did not lead to obvious microstructural changes. The results were consistent with the previous findings that TG had a stronger effect on improving gel quality and reducing water mobility.

### 2.6. Protein Cross-Linking Network Formation Revealed by SDS-PAGE Analysis

**Figure 7:**
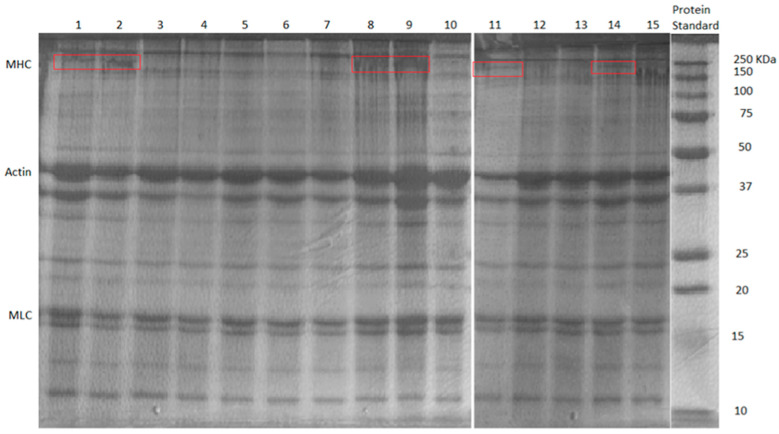
The SDS-PAGE results of BBD treatments FSC surimi gel. Red rectangle represents the MHC bands (~200 kDa).

Figure 7 shows the results of the SDS-PAGE test. The myosin heavy chain (MHC) bands settled around 200 kDa [38]. Samples from BBD treatments 1, 2, 9, 11, and 14 displayed the MHC band (circled by the red rectangles). The commonality among these samples was that they all had less TG wt% (i.e., 0.1/0.5%). Apparently, with the low dosage of TG used in these samples, myosin from the FSC mince did not all form into cross-links (well-formed cross-linked MHCs would be water-insoluble and could not move through the gel during the electrophoresis to form the MHC band [38]). The gel samples showing MHC bands also had poor gel strength and textural properties, and SEM images showed them to have loosely formed network microstructures. It should be noted that all samples showed bands for actin and myosin light chain (MLC), as they were more stable, and it was more difficult to form cross-links between them [38]. The MHCs appeared to be directly responsible for forming cross-links between myosin molecules. The effects of MMF and CLG were not obvious in the SDS-PAGE test for BBD treatments, indicating that they were not directly involved in the formation of myofibrillar protein cross-links.

### 2.7. Synergistic Effects of Additives Revealed by Response-Surface Modeling and Optimization of the Process

The response-surface models (RSMs) used to evaluate the effects of the combination of MMF (A), TG (B), and CLG (C) levels on the estimated functions for gel strength (Y_1_), hardness (Y_2_), springiness (Y_3_), cohesiveness (Y_4_), and resilience (Y_5_) were established using RStudio, and the resulting quadratic correlations for all Y variables are as follows:

Y_1_ = 0.226 − 0.214 × A − 0.081× B − 0.066 × C + 0.020 × A × B + 0.006 × A × C + 0.003 × B × C + 0.174 × A^2^ + 0.092 × B^2^ + 0.010 × C^2^, (1)

Y_2_ = 412 − 252 × A + 60 × B − 116 × C − 118 × A × B + 15 × A × C + 2 × B × C + 251× A^2^ + 57 × B^2^ + 19 × C^2^, (2)

Y_3_ = 0.725 + 0.019 × A + 0.416 × B + 0.056 × C + 0.06 × A × B − 0.001 × A × C + 0.004 × B × C − 0.064 × A^2^ − 0.327 × B^2^ − 0.009 × C^2^, (3)

Y_4_ = 1.18 − 0.40 × A − 0.66 × B − 0.29 × C + 0.04 × A × B − 0.003 × A × C + 0.02 × B × C + 0.34 × A^2^ + 0.44 × B^2^ + 0.045 × C^2^, (4)

Y_5_ = 0.34 + 0.19 × A + 0.15 × B − 0.05 × C − 0.03 × A × B − 0.03 × A × C + 0.01 × B × C − 0.07 × A^2^ − 0.09 × B^2^ + 0.01 × C^2^. (5)

Table 4 shows the statistical analysis for these estimated functions. The *p*-values based on the F-statistical test were less than 0.05 for all equations, indicating that these models were all significant. The R-squares of hardness and springiness were above 0.7, suggesting the fitting of the quadratic model to the data was reasonably good. The R-squares of gel strength, cohesiveness, and resilience were lower than 0.7, revealing that the quadratic models might not fit the data very well. As shown in Table 1, the hardness and springiness data showed more significant differences among the samples with different treatments. The fitting of the models to these datasets was expected to be better. While gel strength, cohesiveness, and resilience data changed much less among the samples with different treatments, the fitting of the model to these datasets was less good as the data covered much smaller ranges. According to the results of the ANOVA test for each factor (Figure S3), MMF and CLG were both significant factors for gel strength as a variable. The CLG, the synergistic effect of MMF × TG, and the synergistic effect of MMF × CLG were significant factors for hardness as a variable. The TG and the synergistic effect of MMF × TG were significant factors for springiness as a variable. The synergistic effect of MMF × CLG was the only significant factor for resilience as a variable. The CLG factor was the only significant factor for cohesiveness as a variable. These results showed that optimization of the additive levels and their synergistic effects is a complex process. Different combinations might be chosen for different objectives. There is no straightforward way to produce the “best” surimi gel from FSC by adjusting the additive levels.

Nonetheless, optimization of the gel-making process can be assisted with these RSM equations. For example, if the goal is to achieve the maximum gel strength, the predicted treatment from the model is 1.0 wt% MMF, 1.0 wt% TG, and 5.0 wt% CLG, corresponding to a gel strength value of 0.182 N-m. These models can guide the adjustment of additives to obtain desired properties, like the appropriate hardness and chewiness, of FSC surimi with various combinations of MMF, TG, and CLG wt%. As the MMF and CLG are more cost-effective and can bring additional health benefits, these models can be utilized to design and produce a surimi gel with less TG usage yet comparable product properties. It should be noted that these models should only be used to predict the values of a variable as a function of the additive levels within a reasonable range. They are not meant to yield accurate predictions for the exact values of the variables. The predicted results would need to be validated when these models are to be applied in practice.

## 3. Conclusions

In this study, the synergistic effect of Manufactured Microfiber (MMF), Transglutaminase (TG), and Chicken Skin Collagen (CLG) on surimi gel from Frozen-stored Silver Carp (FSC) was observed using a gel strength test, a textural profile analysis, an SAOS rheological test, an FT-IR test, a WHC test, a water mobility test, microstructure images, and an SDS-PAGE test. A quadratic model with the Box–Behnken Design (BBD) was used to estimate the synergistic effects. TG showed a more obvious effect than MMF and CLG in these tests. However, CLG and MMF have considerable value, like increasing WHC, higher cost-effectiveness, and health benefits. Increasing CLG and MMF wt% in the recipe can achieve a similar effect with less TG wt% by the estimated response-surface model. The improvement of gel quality of FSC with the synergistic effect of MMF, TG, and CLG indicated the potential to utilize FSC as food resources and enhance economic incentives for Silver Carp capturing.

## 4. Materials and Methods

### 4.1. Materials

The fresh Silver Carp fillets with skin and bones (Figure S4) were purchased from Two Rivers Fisheries, Inc. in Wickliffe, KY, USA. The fillets were stored at −20 °C in the freezer. The MMF was obtained from the University of Maine, Maine, USA. The food-grade TG (100–120 U/g) was purchased from Modernist Pantry, Eliot, ME, USA. The food-grade TG was used because it was cheaper than purified TG enzyme and is widely used in the food industry. Essentia Protein Solutions, Ankeny, IA, USA, produced the naturally dehydrated chicken broth (collagen). The NaCl was purchased from Fisher Scientific, Waltham, MA, USA. The 30% Acrylamide/bis solution was made with 29.2 g of acrylamide (Fisher BioReagents, Pittsburgh, PA, USA) and 0.8 g of bis-acrylamide (Fisher BioReagents, Pittsburgh, PA, USA) per 100 mL solution with deionized water. The 1.5 M Tris-HCl (pH 8.8) solution was made with 18.15 g of Tris-base (Fisher Scientific, Waltham, MA, USA) and around 60 mL of deionized water, adjusted to pH 8.8 with 6 N HCl (Fisher Scientific, Waltham, MA, USA), and filled to 100 mL with deionized water. The 0.5 M Tris-HCl (pH 6.8) solution was made with 6.0 g of Tris-base and around 60 mL of deionized water, adjusted to pH 8.8 with 6 N HCl, and filled to 100 mL with deionized water. The 10% SDS solution was made with 10.0 g of SDS (Fisher BioReagents, Pittsburgh, PA, USA) and deionized water for each 100 mL solution. TEMED, Ammonium persulphate (APS), and standard protein ladder were purchased from Bio-Rad Laboratories, Inc., Hercules, CA, USA. Tris-Glycine-SDS running buffer was made with 30.0 g of Tris-base, 144.0 g of glycine (National Diagnostics, Atlanta, GA, USA), and 10.0 g of SDS with deionized water per 1000 mL buffer. The sample buffer was made with 1.0 mL of 0.5 M Tris-HCl (pH 6.8), 2.0 mL of 25% Glycerol (Carolina Biological Supply, Burlington, NC, USA), 0.08 mL of 0.05% Bromophenol blue (Fisher Scientific, Waltham, MA, USA), 1.6 mL of 10% SDS, 2.92 mL of deionized water, and 0.4 mL of β-mercaptoethanol (Sigma-Aldrich, Burlington, MA, USA). All the chemicals used in this study were of analytical grade.

### 4.2. Making of the Surimi Gel

This surimi-making method was adjusted from the method of Zhang’s group [31]. The Frozen Silver Carp (FSC) fillets were thawed at 5 °C in the refrigerator and then cut into 4–5 cm pieces to remove bones and skin. The deboned fillet pieces were washed 3–4 times with 5 °C water to remove additional blood and soluble remains. A gauze was used to remove surface water on washed FSC. The moisture content of FSC was 78–80% before use. Then, the washed FSC dorsal muscle was taken and put into a meat blender (Oster 2130489 3 Cup Mini Food Chopper, Sunbeam Products, Inc., Boca Raton, FL, USA). The meat blender was placed in an ice bucket to maintain a low temperature (<10 °C). The fillet pieces were blended with 2.5 wt% NaCl for 3 min to make FSC mince. Three levels (low, medium, and high) were used for each of the additives. The actual wt% of each level for the additives is listed in Table 5. The TG levels were 0.1–1.0 wt% because the recipe of the food-grade Transglutaminase (Modernist Pantry, Eliot, ME, USA) recommended 0.75–1.0 wt% in the total recipe. The MMF levels used were the same as TGs to help compare the effects individually and synergistically. The recommended collagen intake for adults was 2.5–15 g per day [39], which was approximately 1.0–5.0 wt% in the 250 g FSC batch. A Box–Behnken design (BBD) was used to investigate the impact of additives as independent variables on the surimi gel. This design was chosen to produce sufficient data for statistical analysis without having to investigate all possible combinations of variables [29]. The levels used for each variable in the BBD are listed in Table 6, and the treatments were applied to each batch (around 250 g of FSC mince per batch). In addition to the Box–Behnken design, the treatment for the MMF-only group (0 wt% MMF, 0.1 wt% MMF, 0.5 wt% MMF, and 1.0 wt% MMF), the TG-only group (0 wt% TG, 0.1 wt% TG, 0.5 wt% TG, and 1.0 wt% TG), and the MMF–TG combination group (0.5 wt% TG, 0.1 wt% MMF with 0.5 wt% TG, 0.5 wt% MMF with 0.5 wt% TG, and 1.0 wt% MMF with 0.5 wt% TG) were also investigated. To mix these ingredients with FSC mince, they were blended in an ice bucket for 2 min using a meat blender. The well-mixed mince was filled into 25 × 30 mm plastic cylinder casings and sealed tightly with plastic film. For each treatment, five replications were produced. These casings were placed into a water bath with a two-step thermal gelation process: heat at 35 °C for 2 h and then at 90 °C for 20 min. This procedure ensured that TG had the best effect on the surimi gel [31]. The formed surimi gel was removed from the casings and stored at 5 °C in the refrigerator. Figure S5 shows one example of our samples and the plastic casing.

### 4.3. Gel Strength Test

The breaking force (N) and the deformation distance (m) were measured by the TA.XT Plus Texture Analyzer (Texture Technologies Corp., Surrey, UK), modified from the method of Zhang’s group [31]. Probe P5 was selected for the test. The pre-test speed was 2.0 mm/s, the test speed was 1.0 mm/s, and the post-test speed was 10 mm/s. The trigger force was 10 g. The compression ratio was set at 50%. The gel strength (Nm) was calculated by the multiplication of the breaking force (N) and the deformation distance (m).

### 4.4. Texture Profile Analysis (TPA) Test

The texture profile properties were also measured by the VERTEX 80v. Hyperion 2000 Infrared Microscope (Texture Technologies Corp., Surrey, UK), modified from the method of Zhang’s group [31]. Probe P50 was selected for this test. The pre-test speed was 2.0 mm/s, the test speed was 1.0 mm/s, and the post-test speed was 10 mm/s. The trigger force was 10 g. The compression ratio was set at 75%. The hardness, cohesiveness, springiness, resilience, and chewiness were obtained from the results.

### 4.5. Rheological Test

The Discovery HR-2 Rheometer (TA Instruments, New Castle, DE, USA) was used for a small-amplitude oscillatory shear (SAOS) test to measure the viscoelastic properties. In this study, the frequency sweep test was set to the same strain (1%) with a frequency range of 0.1 to 100 Hz based on the method of Xie’s group [38]. The samples were cut into 1 mm thickness and placed on a parallel plate (diameter = 40 mm). The results of the MMF-only group, the TG-only group, and the MMF–TG combination group were recorded for this test.

### 4.6. FT-IR Test

The surimi samples from the MMF-only, TG-only, and MMF–TG combination groups were detected using the Thermo FT-IR Microscope (Bruker Corporation, Billerica, MA, USA) with some modifications from Wei’s group [37]. The samples were cut into 1 mm thick circles and freeze-dried at −20 °C for 72 h. The FT-IR data were recorded with 16 scans in the wavenumber range of 4000~400 cm^−1^, and the resolution was 4 cm^−1^. The raw data were accessed using OPUS software (Version 7.0, Bruker Corporation, Billerica, MA, USA) and normalized before the analysis.

### 4.7. LF-NMR Test

A low-field nuclear magnetic resonance (LF-NMR) spectrum (MesoMR23-060V-1, Niumai Analytical Instrument Co., Shanghai, China) was used to measure the water mobility for samples from the BBD experiments. The procedure and test settings were modified from the method of Xie’s group [38]. The magnetic strength was 0.5 T. The resonance frequency was 21 MHz. The test parameters were set as follows: SEQ = CPMG, SW = 200.0 KHz, RFD = 0.080 ms, RG1 = 10.0 db, P1 = 34.0 us, DRG1 = 3, PRG = 1, TW = 5000.0 ms, NS = 8, P2 = 64.0 us, TE = 0.30 ms, and NECH = 6000. The water on the sample surface was wiped before the test. The spin–spin relaxation time (T2) and peak intensity (peak area) were recorded for each sample.

### 4.8. Water Holding Capacity (WHC) Analysis

The water-holding capacity (WHC) is defined as the percentage of water the surimi retains after a constant force is applied [21] with respect to the dry mass of the surimi. The percentage of the original water content of each sample (W.C.) was obtained by the oven-heating method. Briefly, samples were cut into 1.0 cm × 1.0 cm × 1.0 cm pieces, and their masses were measured before heating (m_1_). After 24 h of heating at 105 °C, their masses (m_2_) were recorded accordingly. The equation of W.C. calculation was as follows:

W.C. = (m_1_ − m_2_)/m_1_ × 100%. (6)

The water loss caused by the applied force was measured with a centrifuge (Hybrid Refrigerated Centrifuge CAX-571, TOMY DIGITAL BIOLOGY Co., Ltd., Tokyo, Japan) based on the method of Xie’s group [38]. The settings were a temperature of 10 °C, centrifugation force of 7510× *g*, and centrifugation time of 15 min. Samples were cut into 1.0 cm × 1.0 cm × 1.0 cm pieces for this measurement. The masses before (m_3_) and after (m_4_) centrifugation were recorded. The equation of WHC calculation was as follows:

WHC = 100% − [(m_3_ − m_4_)/(W.C. × m_3_)] × 100%. (7)

Here, W.C. × m_3_ is the water content before centrifuge. m_3_−m_4_ calculates the water being removed by centrifugation; hence, the term in the square brackets calculates the % of water being removed with respect to the dry mass of the sample. The WHC results of samples of the BBD experiments were recorded.

### 4.9. Microstructure Analysis

SEM images of the samples from the BBD experiments were acquired based on the method of Xie’s group [38]. Briefly, samples were cut into 5 mm × 5 mm × 1 mm and fixed to prevent structural changes during freeze-drying. They were placed into a freeze-dryer (SCIENTZ-10ND, Ningbo Xinzhi Freeze-dryer Equipment Co., Ltd., Ningbo, China) at −20 °C for 72 h. The freeze-dried samples were coated with gold and scanned under a scanning electron microscope (JSM-7800F, JEOL Ltd., AkishimaShi, Japan). The SEM images of each sample were photographed during the scanning.

Optical images of the MMF-only, TG-only, and MMF–TG combination group samples were also acquired following the procedure modified from the method of Dong’s group [40]. Briefly, samples were cut into 0.8 cm × 0.8 cm × 1 cm pieces. A low-temperature slicing machine (CM1950, Leica, Germany) was used to freeze the samples at −30 °C and slice them into 10 μm thick pieces. Then, these pieces were stained with a red stain solution and rinsed with deionized water. Optical microscopic images of the samples were recorded with the Olympus BX51 optical light microscope (BX51, Olympus Inc., Tokyo, Japan). The pore areas of microscopic images were then analyzed using ImageJ software (Version 1.45d, National Institutes of Health, Bethesda, MD, USA). Pore area analysis was conducted with binary-processed microscopic images and the “Analyze Particles” process to compute the total pore area, the number of pores, and the average pore size.

### 4.10. SDS-PAGE Analysis

SDS-PAGE (sodium dodecyl sulfate-polyacrylamide gel electrophoresis) was used to analyze the protein composition of the various surimi samples based on the method originally reported by Laemmli et al. [41] and Zhang et al. [42] with some modifications. The samples from the BBD experiments were weighed into 0.1 g pieces and placed in 15 mL centrifuge tubes with 2 mL of the sample buffer. The centrifuge tubes were placed into a boiling water bath for 15 min. Then, the non-soluble surimi was removed by centrifugation at 3000× *g* for 5 min. The 10 µL sample solution was injected into each well of the SDS-PAGE gel. The guide for making the SDS-PAGE gel was taken from the Bio-Red SDS-PAGE Protocol (Bio-Rad Laboratories, Inc., Hercules, CA, USA) with minor modifications. SDS-PAGE was made with 4% stacking gel and 12% resolving gel in a Criterion Cell (Bio-Rad Laboratories, Inc., Hercules, CA, USA). The stacking gel contained 2.0 mL of 30% Acrylamide/bis, 3.75 mL of 0.5 M Tris-HCl (pH 6.8), 9.0 mL of distilled deionized water, 150 µL of 10% SDS, 15 µL of TEMED, and 75 µL of 10% APS. The resolving gel contained 6.0 mL of 30% Acrylamide/bis, 3.75 mL of 1.5 M Tris-HCl (pH 8.8), 5.0 mL of distilled deionized water, 150 µL of 10% SDS, 7.5 µL of TEMED, and 75 µL of 10% APS. The SDS-PAGE gel was placed in the tank connected to an electrophoresis device (Powerpac High-Voltage Power Supply Electrophoresis, Bio-Rad Laboratories, Inc., Hercules, CA, USA). The running buffer filled the tank until it reached the required mark. The electrophoresis device was operated at 200 V for 50 min. The gel, after electrophoresis, was soaked with distilled deionized water for 10 min. Then, the water was poured out, and the gel was covered with a stain solution (Bio-Rad Laboratories, Inc., Hercules, CA, USA) for 3 h. The stained gel was washed with distilled deionized water until the extra stain solution was removed.

### 4.11. Response-Surface Modeling

The data obtained from gel strength tests, TPA tests, and WHC tests were used to construct quadratic polynomial functions with Response-Surface Modeling (RSM) for the optimization of parameters (e.g., levels of additives). The statistical results were calculated using RStudio (Version 2023.06.0 Build 421, Posit Software, Boston, MA, USA). The RStudio code (accessed on 22 May 2024) is available below: https://minhaskamal.github.io/DownGit/#/home?url=https://github.com/JingyiY99/RSM-code/blob/main/MTG_RSM.R.

### 4.12. Data Analysis and Statistical Tests

SPSS software (SPSS, version 16.0, Chicago, IL, USA) and RStudio were used to analyze the data. The data were displayed as mean ± standard deviation (SD). Tukey’s honestly significant difference (Tukey’s HSD) test was used to determine the significant differences among datasets. The *p*-value was set to *p* < 0.05.

## Figures and Tables

**Table 1:** Experimental matrix and observed gel strength and TPA results from randomized runs in the BBD for FSC surimi.

Run	Independent Variables			Dependent Variables				
	MMF (wt%)	TG (wt%)	Collagen (wt%)	Gel Strength (N-m)	Hardness (N)	Resilience	Cohesiveness	Springiness
				(mean ± SD, *n* = 4)	(mean ± SD, *n* = 4)	(mean ± SD, *n* = 4)	(mean ± SD, *n* = 4)	(mean ± SD, *n* = 4)
1	0.1	0.1	3	0.092 ± 0.007 ^a^	240.09 ± 10.39 ^a^	0.3742 ± 0.0098 ^a^	0.6829 ± 0.0197 ^c^	0.8485 ± 0.0293 ^abcd^
2	1	0.1	3	0.099 ± 0.007 ^abc^	279.57 ± 7.68 ^abcd^	0.3644 ± 0.0049 ^a^	0.6705 ± 0.0126 ^c^	0.8172 ± 0.0160 ^ab^
3	0.1	1	3	0.122 ± 0.006 ^bcde^	314.38 ± 47.53 ^bcde^	0.3599 ± 0.0278 ^a^	0.4619 ± 0.0339 ^a^	0.9166 ± 0.0098 ^e^
4	1	1	3	0.144 ± 0.021 ^e^	266.55 ± 23.39 ^abc^	0.3430 ± 0.0654 ^a^	0.5027 ± 0.0998 ^ab^	0.9337 ± 0.0455 ^e^
5	0.1	0.5	1	0.135 ± 0.009 ^de^	329.51 ± 10.16 ^def^	0.3458 ± 0.0284 ^a^	0.7316 ± 0.0597 ^c^	0.9112 ± 0.0250 ^de^
6	1	0.5	1	0.124 ± 0.014 ^cde^	320.52 ± 13.50 ^def^	0.4381 ± 0.0042 ^b^	0.7002 ± 0.0289 ^c^	0.8816 ± 0.0103 ^cde^
7	0.1	0.5	5	0.133 ± 0.010 ^de^	322.53 ± 39.82 ^def^	0.4537 ± 0.0107 ^b^	0.6520 ± 0.1089 ^c^	0.9277 ± 0.0261 ^e^
8	1	0.5	5	0.143 ± 0.014 ^e^	368.58 ± 15.27 ^f^	0.4374 ± 0.0072 ^b^	0.6191 ± 0.0348 ^bc^	0.8958 ± 0.0241 ^de^
9	0.5	0.1	1	0.109 ± 0.011 ^abcd^	244.70 ± 11.59 ^a^	0.3675 ± 0.0011 ^a^	0.6834 ± 0.0036 ^c^	0.8051 ± 0.0279 ^a^
10	0.5	1	1	0.138 ± 0.006 ^de^	332.46 ± 15.92 ^def^	0.4625 ± 0.0255 ^b^	0.6780 ± 0.0577 ^c^	0.8868 ± 0.0186 ^de^
11	0.5	0.1	5	0.093 ± 0.012 ^ab^	262.64 ± 10.39 ^ab^	0.3581 ± 0.0108 ^a^	0.6756 ± 0.0179 ^c^	0.8210 ± 0.0317 ^abc^
12	0.5	1	5	0.136 ± 0.020 ^de^	358.17 ± 15.59 ^ef^	0.4783 ± 0.0057 ^b^	0.7283 ± 0.0399 ^c^	0.9166 ± 0.0089 ^e^
13	0.5	0.5	3	0.118 ± 0.009 ^abcde^	314.21 ± 23.40 ^bcde^	0.4364 ± 0.0096 ^b^	0.7322 ± 0.0232 ^c^	0.8828 ± 0.0391 ^cde^
14	0.5	0.5	3	0.127 ± 0.012 ^cde^	312.07 ± 6.52 ^bcde^	0.4412 ± 0.0165 ^b^	0.7222 ± 0.0613 ^c^	0.8791 ± 0.0194 ^bcde^
15	0.5	0.5	3	0.122 ± 0.006 ^bcde^	316.99 ± 15.60 ^cdef^	0.4332 ± 0.0095 ^b^	0.7307 ± 0.0230 ^c^	0.8971 ± 0.0031 ^de^

Different lowercase letters in a column indicate significant differences (*p* < 0.05) between treatments.

**Table 2:** Experimental matrix and LF-NMR results from randomized runs in the BBD.

Run	Independent Variables			Dependent Variables					
	MMF (wt%)	TG (wt%)	CLG (wt%)	T_21_ (ms)	T_22_ (ms)	T_23_ (ms)	P_1_ (%)	P_2_ (%)	P_3_ (%)
1	0.1	0.1	3	0.606 ± 0.078 ^bcdef^	58.05 ± 0.00 ^c^	589.1 ± 25.91 ^bc^	2.565 ± 0.256 ^a^	93.87 ± 0.699 ^ab^	3.570 ± 0.758 ^bc^
2	1	0.1	3	0.594 ± 0.042 ^bcdef^	58.05 ± 0.00 ^c^	608.0 ± 0.000 ^cd^	2.383 ± 0.180 ^a^	94.49 ± 0.313 ^bc^	3.124 ± 0.425 ^ab^
3	0.1	1	3	0.567 ± 0.059 ^abcde^	53.61 ± 3.07 ^a^	692.7 ± 30.47 ^fg^	2.614 ± 0.320 ^a^	92.69 ± 1.767 ^a^	4.693 ± 1.703 ^c^
4	1	1	3	0.534 ± 0.082 ^abcd^	54.44 ± 2.02 ^ab^	649.1 ± 22.94 ^de^	2.832 ± 0.478 ^a^	94.11 ± 0.373 ^bc^	3.058 ± 0.248 ^ab^
5	0.1	0.5	1	0.446 ± 0.062 ^a^	62.95 ± 0.00 ^d^	775.3 ± 0.000 ^h^	2.696 ± 0.168 ^a^	94.58 ± 0.189 ^bc^	2.719 ± 0.098 ^ab^
6	1	0.5	1	0.496 ± 0.018 ^ab^	58.05 ± 0.00 ^c^	670.4 ± 24.88 ^ef^	2.473 ± 0.233 ^a^	95.02 ± 0.189 ^bc^	2.504 ± 0.122 ^ab^
7	0.1	0.5	5	0.699 ± 0.049 ^efg^	58.05 ± 0.00 ^c^	589.1 ± 25.91 ^bc^	2.517 ± 1.089 ^a^	94.87 ± 1.080 ^bc^	2.612 ± 0.064 ^ab^
8	1	0.5	5	0.680 ± 0.103 ^defg^	53.54 ± 0.00 ^a^	560.7 ± 0.000 ^b^	2.931 ± 0.320 ^a^	94.82 ± 0.210 ^bc^	2.246 ± 0.206 ^a^
9	0.5	0.1	1	0.591 ± 0.106 ^abcdef^	62.95 ± 0.00 ^d^	727.0 ± 26.97 ^g^	2.428 ± 0.297 ^a^	95.37 ± 0.247 ^c^	2.205 ± 0.079 ^a^
10	0.5	1	1	0.513 ± 0.022 ^abc^	58.05 ± 0.00 ^c^	714.9 ± 0.000 ^g^	2.781 ± 0.258 ^a^	94.84 ± 0.317 ^bc^	2.380 ± 0.102 ^a^
11	0.5	0.1	5	0.772 ± 0.084 ^g^	58.05 ± 0.00 ^c^	517.1 ± 0.000 ^a^	2.432 ± 0.100 ^a^	95.24 ± 0.198 ^c^	2.328 ± 0.137 ^a^
12	0.5	1	5	0.645 ± 0.060 ^cdefg^	56.25 ± 2.47 ^bc^	598.6 ± 21.16 ^bc^	2.805 ± 0.244 ^a^	94.82 ± 0.348 ^bc^	2.374 ± 0.115 ^a^
13	0.5	0.5	3	0.665 ± 0.046 ^defg^	58.05 ± 0.00 ^c^	608.0 ± 0.000 ^cd^	2.478 ± 0.163 ^a^	94.90 ± 0.217 ^bc^	2.625 ± 0.138 ^ab^
14	0.5	0.5	3	0.584 ± 0.039 ^abcdef^	58.05 ± 0.00 ^c^	618.3 ± 22.94 ^cd^	2.708 ± 0.277 ^a^	95.11 ± 0.248 ^bc^	2.180 ± 0.079 ^a^
15	0.5	0.5	3	0.723 ± 0.053 ^fg^	58.05 ± 0.00 ^c^	608.0 ± 0.000 ^cd^	2.757 ± 0.253 ^a^	95.09 ± 0.287 ^bc^	2.157 ± 0.104 ^a^

Each value is expressed as means ± S.D. (*n* = 5); different lowercase letters in a column indicate significant differences (*p* < 0.05) between treatments.

**Table 3:** Pore area analysis with WHC results according to optical microscopic images of FSC samples.

	Control	1.0 wt% MMF	0.5 wt% TG	0.5 wt% TG 0.5 wt% MMF	0.5 wt% TG 1.0 wt% MMF
Pore Count	209 ± 8 ^a^	315 ± 25 ^ab^	962 ± 180 ^c^	882 ± 108 ^c^	482 ± 49 ^b^
Total Pore Area (mm^2^)	0.99 ± 0.04 ^a^	0.77 ± 0.02 ^b^	0.75 ± 0.03 ^b^	0.70 ± 0.02 ^b^	0.77 ± 0.09 ^b^
Average Pore Size (μm^2^)	4730 ± 21 ^d^	2465 ± 187 ^c^	796 ± 128 ^a^	806 ± 123 ^a^	1600 ± 118 ^b^
WHC (%)	69.17 ± 1.73 ^ab^	69.07 ± 0.99 ^a^	73.12 ± 0.70 ^ab^	73.18 ± 2.72 ^ab^	73.26 ± 0.49 ^b^

Each value is expressed as means ± S.D. (*n* = 3); different lowercase letters in a row indicate significant differences (*p* < 0.05) between treatments.

**Table 4:** Statistical analysis results of quadratic model for FSC surimi.

Quadratic Model	R^2^	Adjusted R^2^	Predicted R^2^	*p*-Value
Gel Strength (Y_1_)	0.6749	0.6164	0.5171	<0.05
Hardness (Y_2_)	0.7402	0.6934	0.6149	<0.05
Springiness (Y_3_)	0.7548	0.7107	0.6454	<0.05
Cohesiveness (Y_4_)	0.5123	0.4245	0.2697	<0.05
Resilience (Y_5_)	0.5652	0.4869	0.3414	<0.05

**Table 5:** Codes (−1, 0, 1) according to MMF, TG, and collagen wt%.

	Levels (Code)		
Independent Variables	Low (−1)	Medium (0)	High (+1)
MMF (wt%)	0.1	0.5	1.0
TG (wt%)	0.1	0.5	1.0
Collagen (wt%)	1	3	5

**Table 6:** The Box–Behnken design for MMF, TG, and collagen treatment by codes (−1, 0, 1). The actual wt% according to codes is shown in Table 5.

Run	Independent Variables		
	MMF	TG	Collagen
1	−1	−1	0
2	1	−1	0
3	−1	1	0
4	1	1	0
5	−1	0	−1
6	1	0	−1
7	−1	0	1
8	1	0	1
9	0	−1	−1
10	0	1	−1
11	0	−1	1
12	0	1	1
13	0	0	0
14	0	0	0
15	0	0	0

## Data Availability

The data presented in this study are available upon request from the corresponding author.

## Supplementary Materials

The following supporting information can be downloaded at: https://www.mdpi.com/article/10.3390/gels10060374/s1, Table S1: Effect of MMF on the gel quality of FSC with various MMF dosages and without MMF (control); Table S2: Effect of TG on the gel quality of FSC with various TG dosages and without TG (control); Table S3: Effect of MMF & TG on the gel quality of FSC with the same TG dosage (0.5 wt%) and various MMF dosages and without MMF (control); Figure S1: Example of binary-processed microscopic image of 0.5 wt% TG sample; Figure S2: SEM images for BBD treatments of FSC surimi; Figure S3: RStudio ANOVA Results; Figure S4: Frozen silver carp fillet with skin and bone; Figure S5: Example of our samples (right) and the plastic casing (left).
