# Supplementary material for: Improvement of Surimi Gel from Frozen-Stored Silver Carp
Source: Gels. 2024 May 28;10(6):374. doi: 10.3390/gels10060374 (PMC11203346; doi:10.3390/gels10060374)

Improvement of Surimi Gel from Frozen-Stored Silver Carp

Supplementary Materials

Jingyi Yang [1], Xiliang Yu [2,3], Xiuping Dong [2,3] and Chenxu Yu [1,*]

[1] Department of Agricultural and Biosystems Engineering, Iowa State University, Ames, IA 50011, USA; jingyiy@iastate.edu
[2] Academy of Food Interdisciplinary Science, School of Food Science and Technology, Dalian Polytechnic University, Dalian 116034, China
[3] National Engineering Research Center of Seafood, Collaborative Innovation Center of Seafood Deep Processing, Liaoning Province Collaborative Innovation Center for Marine Food Deep Processing, Dalian 116034, China
* Correspondence: chenxuyu@iastate.edu; Tel.: +1-515-294-4554

Table of Contents

Table S1. -- 3

Table S2. -- 3

Table S3. -- 3

Figure S1. --- 4

Figure S2. --- 5-6

Figure S3. --- 7-8

Figure S4. --- 9

Figure S5. --- 9

Table S1. Effect of MMF on the gel quality of FSC with various MMF dosages and without MMF (control).

	Control	0.1 wt% MMF	0.5 wt% MMF	1.0 wt% MMF
Breaking Force(N)	1.40 ± 0.13^{a}	1.60 ± 0.18^{ab}	1.97 ± 0.26^{bc}	2.38 ± 0.30^{c}
Deformation(m)	0.0075 ± 0.0007^{a}	0.0092 ± 0.0011^{a}	0.0091 ± 0.0010^{a}	0.0087 ± 0.0012^{a}
Gel Strength (N-m)	0.0105 ± 0.0003^{a}	0.0148 ± 0.0027^{ab}	0.0182 ± 0.0044^{b}	0.0209 ± 0.0049^{b}
Hardness(N)	42.0 ± 6.5^{ab}	34.5 ± 3.5^{a}	54.5± 9.8^{b}	48.9 ± 10.0^{b}
Cohesiveness	0.29 ± 0.02^{a}	0.24 ± 0.03^{a}	0.29 ± 0.02^{a}	0.28 ± 0.04^{a}
Springiness	0.78 ± 0.04^{a}	0.70 ± 0.10^{a}	0.74 ± 0.06^{a}	0.67 ± 0.05^{a}
Chewiness(N)	9.58 ± 1.96^{ab}	6.02 ± 1.72^{a}	11.9 ± 3.72^{b}	9.14 ± 2.98^{a}
Resilience	0.087 ± 0.003^{a}	0.073 ± 0.005^{a}	0.085 ± 0.014^{a}	0.079 ± 0.010^{a}

Each value is expressed as means ± S.D. (*n*=5); Different lowercase letters in a row indicate significant differences ($p < 0.05$) between treatments.

Table S2. Effect of TG on the gel quality of FSC with various TG dosages and without TG (control).

	Control	0.1 wt% TG	0.5 wt% TG	1.0 wt% TG
Breaking Force(N)	1.03± 0.11^{a}	1.89 ± 0.05^{b}	2.36 ± 0.16^{c}	3.49 ± 0.24^{d}
Deformation(m)	0.0098 ± 0.0006^{a}	0.0107 ± 0.0010^{a}	0.0107 ± 0.0007^{a}	0.0110 ± 0.0004^{a}
Gel Strength (N-m)	0.0101 ± 0.0010^{a}	0.0202 ± 0.0022^{b}	0.0254 ± 0.0034^{b}	0.0383 ± 0.0033^{c}
Hardness(N)	22.8 ± 3.5^{a}	42.6 ± 2.3^{b}	37.1 ± 2.8^{b}	69.8 ± 2.8^{c}
Cohesiveness	0.159 ± 0.017^{a}	0.224 ± 0.023^{b}	0.251 ± 0.019^{b}	0.228 ± 0.025^{b}
Springiness	0.777 ± 0.059^{a}	0.779 ± 0.173^{a}	0.783 ± 0.014^{a}	0.789 ± 0.177^{a}
Chewiness(N)	2.12 ± 0.54^{a}	10.1 ± 2.6^{b}	7.31 ± 1.06^{b}	16.4 ± 1.9^{c}
Resilience	0.050 ± 0.002^{a}	0.048 ± 0.48^{a}	0.082 ± 0.006^{a}	0.042 ± 0.43^{a}

Each value is expressed as means ± S.D. (*n*=5); Different lowercase letters in a row indicate significant differences ($p < 0.05$) between treatments.

Table S3. Effect of MMF & TG on the gel quality of FSC with the same TG dosage (0.5 wt%) and various MMF dosages and without MMF (control).

	0.5 wt% TG	0.5 wt% TG<br>0.1 wt% MMF	0.5 wt% TG<br>0.5 wt% MMF	0.5 wt% TG<br>1.0 wt% MMF
Breaking Force(N)	2.36 ± 0.16^{a}	3.44 ± 0.20^{b}	3.88 ± 0.24^{c}	4.21 ± 0.14^{c}
Deformation(m)	0.0107 ± 0.0007^{a}	0.0118 ± 0.0005^{a}	0.0114 ± 0.0006^{a}	0.0118 ± 0.0006^{a}
Gel Strength(N-m)	0.0254 ± 0.0034^{a}	0.0406 ± 0.0038^{b}	0.0443 ± 0.0045^{b}	0.0498 ± 0.0037^{c}
Hardness(N)	37.1 ± 2.8^{a}	71.4 ± 4.7^{b}	81.7 ± 6.0^{c}	77.6 ± 3.2^{bc}
Cohesiveness	0.251 ± 0.019^{a}	0.228 ± 0.039^{a}	0.272 ± 0.016^{a}	0.262 ± 0.018^{a}
Springiness	0.783 ± 0.014^{a}	0.826 ± 0.142^{a}	0.870 ± 0.017^{a}	0.851 ± 0.014^{a}
Chewiness(N)	7.31 ± 1.06^{a}	18.1 ± 2.7^{b}	19.4 ± 2.27^{b}	17.4 ± 1.74^{b}
Resilience	0.082 ± 0.006^{a}	0.093 ± 0.325^{a}	0.101 ± 0.004^{a}	0.097 ± 0.002^{a}

Each value is expressed as means ± S.D. (*n*=5); Different lowercase letters in a row indicate significant differences ($p < 0.05$) between treatments.

Figure S1. Example of binary-processed microscopic image of 0.5 wt% TG sample.

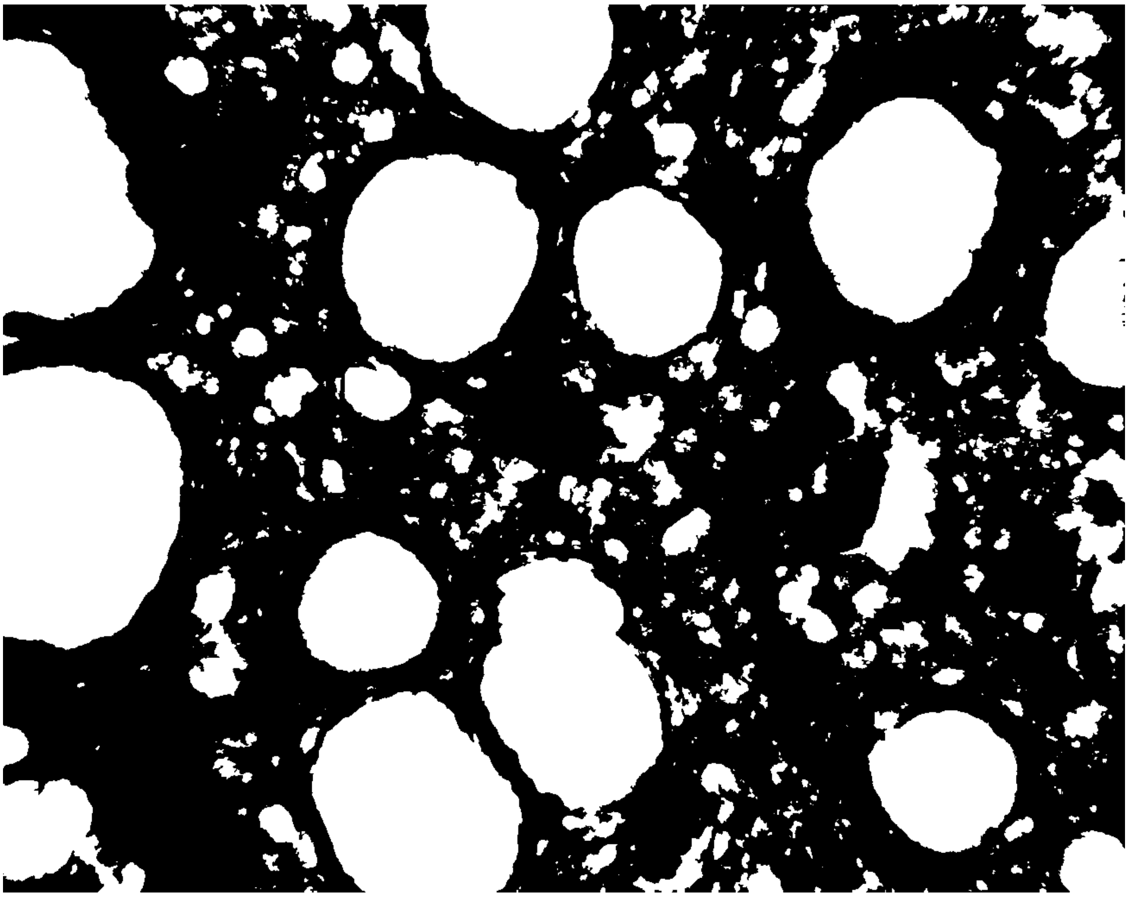

Figure S2. SEM images for BBD treatments of FSC surimi

Observation	MMF wt%	TG wt%	CLG wt%	200×	1000×
1	0.1	0.1	3	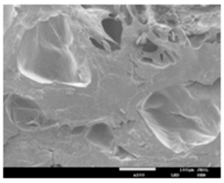	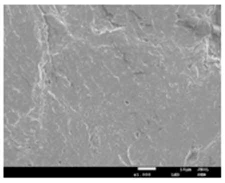
2	1	0.1	3	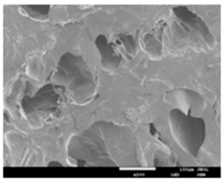	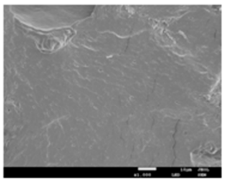
3	0.1	1	3	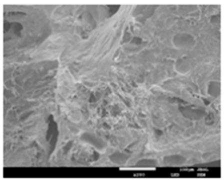	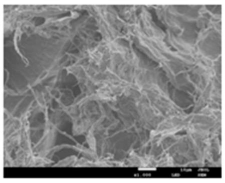
4	1	1	3	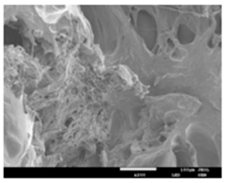	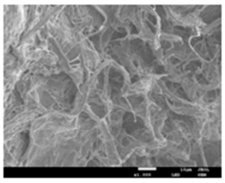
5	0.1	0.5	1	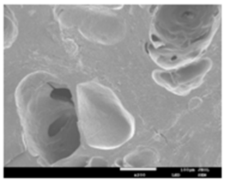	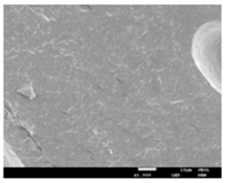
6	1	0.5	1	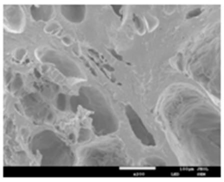	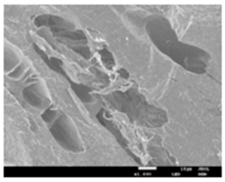
7	0.1	0.5	5	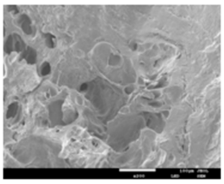	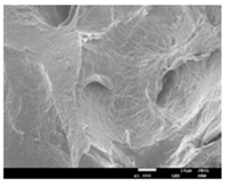
8	1	0.5	5	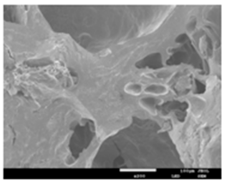	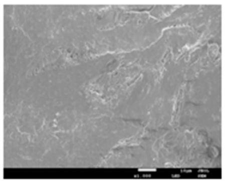

Figure S2. SEM images for BBD treatments of FSC surimi Continue

Observation	MMF wt%	TG wt%	CLG wt%	200×	1000×
9	0.5	0.1	1	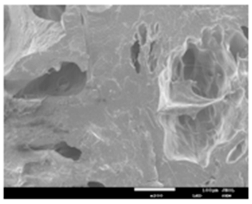	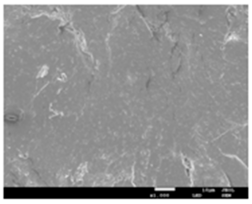
10	0.5	1	1	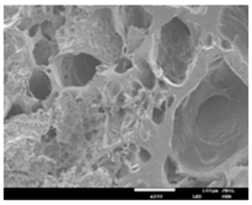	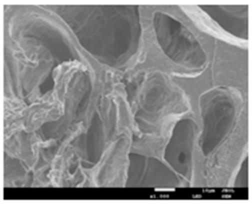
11	0.5	0.1	5	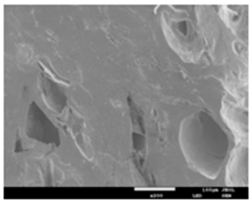	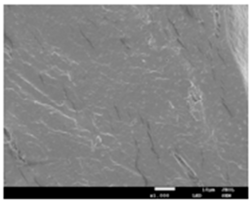
12	0.5	1	5	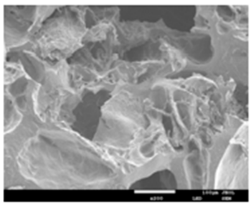	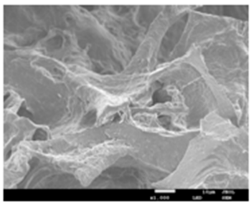
13	0.5	0.5	3	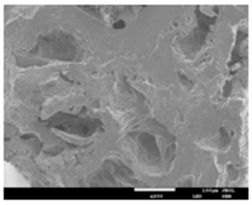	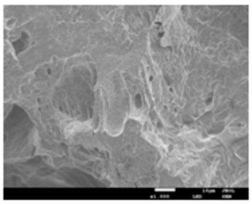
14	0.5	0.5	3	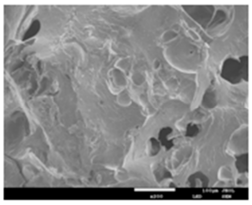	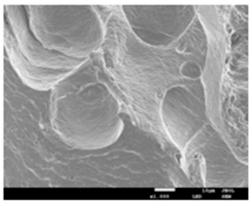
15	0.5	0.5	3	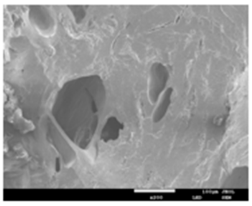	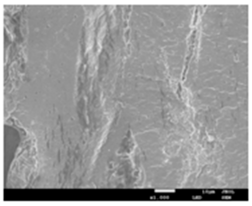

Figure S3. RStudio ANOVA Results

(a) Gel Strength:

```
Residuals:
       Min         1Q     Median         3Q        Max 
-0.0215044 -0.0086878 -0.0001213  0.0078207  0.0270529 

Coefficients:
             Estimate Std. Error t value Pr(>|t|)    
(Intercept)  0.225838   0.050200   4.499 4.09e-05 ***
MMF         -0.213515   0.076022  -2.809  0.00708 ** 
TG          -0.080941   0.076022  -1.065  0.29212    
CLG         -0.065695   0.024338  -2.699  0.00945 ** 
I(MMF^2)     0.173838   0.067540   2.574  0.01307 *  
I(TG^2)      0.092279   0.067540   1.366  0.17796    
I(CLG^2)     0.010096   0.003943   2.560  0.01353 *  
MMF:TG       0.019723   0.014962   1.318  0.19344    
MMF:CLG      0.006494   0.003377   1.923  0.06017 .  
TG:CLG       0.003255   0.003377   0.964  0.33973    
---
Signif. codes:  0 '***' 0.001 '**' 0.01 '*' 0.05 '.' 0.1 ' ' 1

Residual standard error: 0.01219 on 50 degrees of freedom
Multiple R-squared:  0.6749,	Adjusted R-squared:  0.6164 
F-statistic: 11.53 on 9 and 50 DF,  p-value: 1.542e-09
```

(b) Hardness:

```
Residuals:
    Min      1Q  Median      3Q     Max 
-57.860  -9.581  -0.088  13.269  46.169 

Coefficients:
            Estimate Std. Error t value Pr(>|t|)    
(Intercept)  411.964     95.504   4.314 7.56e-05 ***
MMF         -252.248    144.629  -1.744 0.087287 .  
TG            59.735    144.629   0.413 0.681355    
CLG         -116.191     46.302  -2.509 0.015378 *  
I(MMF^2)     251.432    128.493   1.957 0.055969 .  
I(TG^2)       57.480    128.493   0.447 0.656563    
I(CLG^2)      18.721      7.502   2.496 0.015922 *  
MMF:TG      -117.828     28.464  -4.140 0.000134 ***
MMF:CLG       15.170      6.424   2.361 0.022142 *  
TG:CLG         2.277      6.424   0.354 0.724467    
---
Signif. codes:  0 '***' 0.001 '**' 0.01 '*' 0.05 '.' 0.1 ' ' 1

Residual standard error: 23.2 on 50 degrees of freedom
Multiple R-squared:  0.7402,	Adjusted R-squared:  0.6934 
F-statistic: 15.83 on 9 and 50 DF,  p-value: 7.698e-12
```

Figure S3. RStudio ANOVA Results Continue.

(c) Springiness

```
Residuals:
      Min        1Q    Median        3Q       Max 
-0.061539 -0.012385  0.003396  0.013473  0.065606 

Coefficients:
              Estimate Std. Error t value Pr(>|t|)    
(Intercept)  0.7245822  0.1006060   7.202  2.9e-09 ***
MMF          0.0185799  0.1523559   0.122  0.90343    
TG           0.4157687  0.1523559   2.729  0.00875 ** 
CLG          0.0561464  0.0487758   1.151  0.25516    
I(MMF^2)    -0.0636731  0.1353571  -0.470  0.64011    
I(TG^2)     -0.3274684  0.1353571  -2.419  0.01923 *  
I(CLG^2)    -0.0088390  0.0079027  -1.118  0.26871    
MMF:TG       0.0627145  0.0299849   2.092  0.04158 *  
MMF:CLG     -0.0008403  0.0067674  -0.124  0.90168    
TG:CLG       0.0040117  0.0067674   0.593  0.55599    
---
Signif. codes:  0 ‘***’ 0.001 ‘**’ 0.01 ‘*’ 0.05 ‘.’ 0.1 ‘ ’ 1

Residual standard error: 0.02444 on 50 degrees of freedom
Multiple R-squared:  0.7548,	Adjusted R-squared:  0.7107 
F-statistic:  17.1 on 9 and 50 DF,  p-value: 1.934e-12
```

(d) Cohesiveness

```
Residuals:
      Min        1Q    Median        3Q       Max 
-0.157625 -0.032700  0.008351  0.048317  0.112687 

Coefficients:
             Estimate Std. Error t value Pr(>|t|)    
(Intercept)  1.178846   0.284084   4.150 0.000129 ***
MMF         -0.398681   0.430212  -0.927 0.358529    
TG          -0.660390   0.430212  -1.535 0.131080    
CLG         -0.285507   0.137730  -2.073 0.043350 *  
I(MMF^2)     0.342797   0.382212   0.897 0.374082    
I(TG^2)      0.442016   0.382212   1.156 0.252985    
I(CLG^2)     0.044977   0.022315   2.016 0.049238 *  
MMF:TG       0.041702   0.084669   0.493 0.624503    
MMF:CLG     -0.003409   0.019109  -0.178 0.859119    
TG:CLG       0.019134   0.019109   1.001 0.321495    
---
Signif. codes:  0 ‘***’ 0.001 ‘**’ 0.01 ‘*’ 0.05 ‘.’ 0.1 ‘ ’ 1

Residual standard error: 0.069 on 50 degrees of freedom
Multiple R-squared:  0.5123,	Adjusted R-squared:  0.4245 
F-statistic: 5.835 on 9 and 50 DF,  p-value: 1.593e-05
```

(d) Resilience

```
Residuals:
      Min        1Q    Median        3Q       Max 
-0.135138 -0.014345  0.003236  0.016389  0.068699 

Coefficients:
             Estimate Std. Error t value Pr(>|t|)   
(Intercept)  0.339136   0.148523   2.283  0.02669 * 
MMF          0.193229   0.224920   0.859  0.39439   
TG           0.149941   0.224920   0.667  0.50807   
CLG         -0.045021   0.072007  -0.625  0.53466   
I(MMF^2)    -0.070236   0.199825  -0.351  0.72670   
I(TG^2)     -0.089805   0.199825  -0.449  0.65507   
I(CLG^2)     0.010711   0.011667   0.918  0.36299   
MMF:TG      -0.032543   0.044266  -0.735  0.46567   
MMF:CLG     -0.028397   0.009991  -2.842  0.00647 **
TG:CLG       0.005263   0.009991   0.527  0.60065   
---
Signif. codes:  0 ‘***’ 0.001 ‘**’ 0.01 ‘*’ 0.05 ‘.’ 0.1 ‘ ’ 1

Residual standard error: 0.03608 on 50 degrees of freedom
Multiple R-squared:  0.5652,	Adjusted R-squared:  0.4869 
F-statistic:  7.22 on 9 and 50 DF,  p-value: 1.242e-06
```

Figure S4. Frozen silver carp fillet with skin and bone (around 18 cm length and 6 cm width).

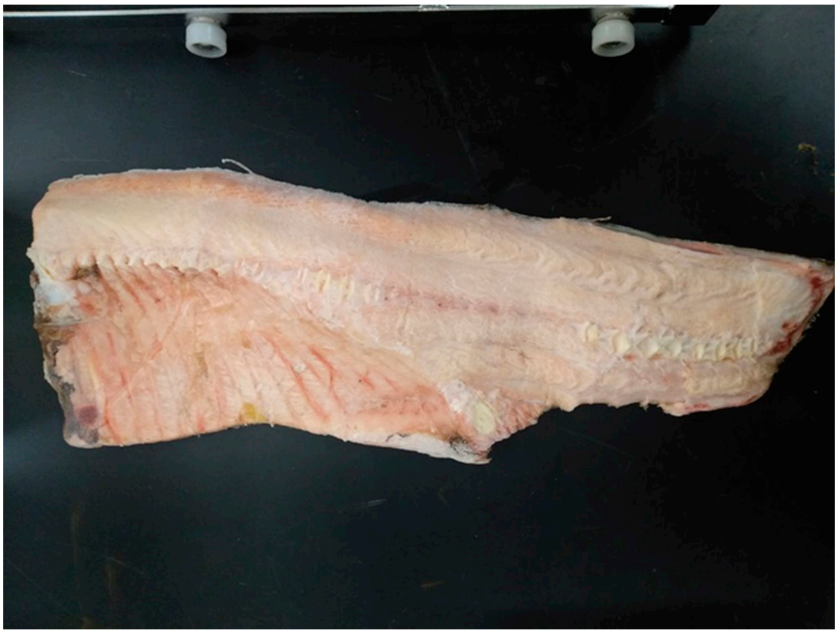

Figure S5. Example of our samples (right) and the plastic casing (left).

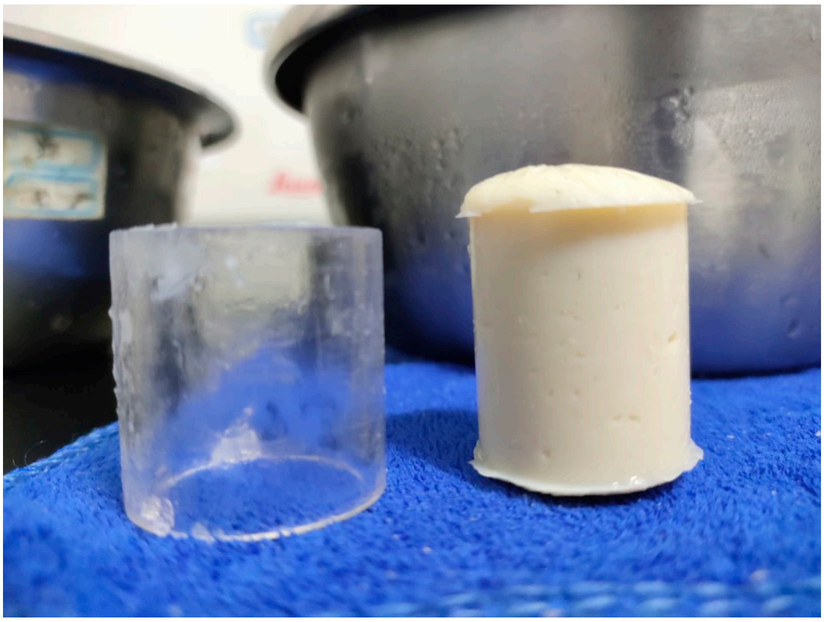

Supplement: Supplementary file 1 [file gels-10-00374-s001.zip › gels-3012964-supplementary.pdf]
